# Artificial intelligence insight on structural basis and small molecule binding niches of NMDA receptor

**DOI:** 10.1016/j.csbj.2025.07.027

**Published:** 2025-07-14

**Authors:** Yunsheng Liu, Han Tang, Jinfang Zhang, Dan Li, Zengwei Kou

**Affiliations:** a Cancer Center, Shenzhen Hospital (Futian) of Guangzhou University of Chinese Medicine, Shenzhen 518000, China; b Department of Neurosurgery, Institute of Translational Medicine, Shenzhen Second People’s Hospital/the First Affiliated Hospital of Shenzhen University Health Science Center, Shenzhen 518035, China; c Department of Neurosurgery, The First Affiliated Hospital, Hengyang Medical School, University of South China, Hengyang 421001, China; d School of Computer and Communication, Jiangsu Vocational College of Electronics and Information, Jiangsu 223003, China; e Department of Laboratory Medicine and Pathobiology, Temerty Faculty of Medicine, University of Toronto, Toronto, ON M5S 1A8, Canada

**Keywords:** Glutamate receptor, Protein prediction, AlphaFold, RoseTTAFold, Protein docking

## Abstract

NMDA receptors are critical to neuronal activity and play essential roles in synaptic transmission, learning, and memory. Despite significant advances in X-ray crystallography and cryo-electron microscopy (cryo-EM), the structural diversity of NMDA receptors across species and the variations among receptor subtypes within the same species remain insufficiently explored. Additionally, several key small molecule binding sites, such as those for agonists, antagonists, and allosteric modulators, have not been fully characterized. In this study, we utilized state-of-the-art artificial intelligence algorithms to model NMDA receptors across multiple species and found that they all adopted a bouquet-like dimer-of-dimer structure. By comparing these models with cryo-EM resolved structures, we assessed the accuracy of the predictions and complemented the structural data with detailed models of transmembrane domain regions, which are traditionally challenging for experimental methods. Furthermore, through the integration of AI-based prediction tools and molecular dynamic simulations, we highlighted potential binding sites for agonists, competitive antagonists, and pore blockers at amino acid resolution. This AI-enhanced approach builds traditional structural biology techniques, revealing that NMDA receptors from different species adopt highly similar three-dimensional architectures, while also exhibiting subtype-specific structural features. Furthermore, our identification of ligand binding pockets at the amino acid resolution provides a more detailed understanding of receptor-ligand interactions, offering potential templates for rational drug design and optimization.

## Graphical Abstract

**Figure:**
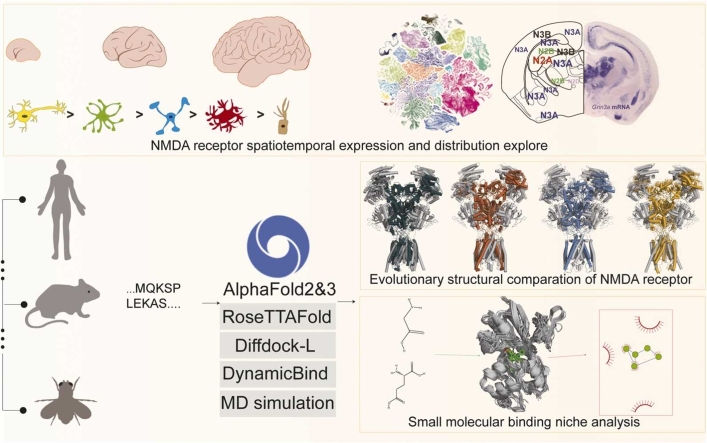

## 1 Introduction

*N*-methyl-*D*-aspartate receptors (NMDARs) are di- or tri- heteromeric ion channels that assembled by two identical glycine-bound GluN1 subunits, and two identical or different alternative glutamate-bound GluN2 subunits (GluN2A-N2D) or glycine-bound GluN3 subunits (GluN3A, N3B) [1], [2]. It’s typically activated after *α*-amino-3-hydroxy-5-methyl-4-isoxazolepropionic acid receptors (AMPARs) and contributes to the later phase of the excitatory postsynaptic potentials (EPSPs) in the postsynaptic area [3], [4]. Activation of NMDARs requires both agonists bound to force the pore open and membrane depolarization to release magnesium blockage [5]. Therefore, NMDARs are often referred to as "coincidence detectors". Upon activation, they allow potassium efflux and sodium and calcium influx [6], which further depolarizes the membrane potential and triggers calcium-related signaling cascades. Consequently, NMDARs play a vital role in numerous brain functions, including synaptic plasticity and neuron-astrocyte communication [3]. Dysfunction, altered expression, or aberrant subcellular distribution of NMDARs are associated with various psychiatric and neurological disorders [7], [8].

NMDARs have been expressed in a variety of organisms early in evolution, with the divergence between pre-NMDARs and pre-non-NMDARs occurring as early as in *Arabidopsis thaliana* and *Ctenophora* [9]. The distinct subtypes of NMDARs began to emerge and differentiate after *Cnidaria* [9]. Given their importance, considerable research has been dedicated to understanding the structure and function of NMDARs. Early studies utilized X-ray crystallography to study isolated domains [10] and domain combinations [11], while more recent advances have focused on resolving receptor structures using single-particle cryo-EM [12] for recombinant receptors and cryo-ET (cryo-electron tomography) for *in situ* receptors [13], [14], [15]. However, substantial functional and structural differences persist among NMDAR subtypes, including domain assembly patterns and ligand activation mechanisms. Moreover, comparative analyses of NMDARs across different species are even more challenging. Additionally, the time and resource demands of cryo-EM and cryo-ET, coupled with their resolution limitations, have hindered high-throughput structure elucidation, and the precise binding sites for some small molecules remain unclear.

In order to obtain the structures of NMDARs in different species for comparison of their intrinsic regularity and validation, we employed state-of-the-art AI-based protein modeling tools, AlphaFold [16], to predict NMDAR structures in humans and other species. AlphaFold, especially the recently released AlphaFold multimer (or AlphaFold2) [17] and AlphaFold3 [16], have the capable of predicting the structure of proteins and protein interactions. We then compared these predicted models with experimentally determined structures and validated the aspects of the apo-state models using disulfide crosslinking experiments.

Based on our predictions, we analyzed the evolutionary patterns of NMDARs across species and highlighted the similarities and differences among human GluN1-N2 NMDAR subtypes. Furthermore, we leveraged advanced protein language models, such as RoseTTAFold All-Atom [18], DiffDock-L [19] and DynamicBind [20], to explore the agonist-binding pocket and blocker-binding cavities in NMDARs. To validate the above predictions and capture the dynamic receptor-small molecule binding structure, we also used molecular dynamic simulations for exploration. Moreover, we also utilized public online single-cell RNA sequencing (sc-RNA) data to map the spatiotemporal expression and distribution of NMDARs in the brain, as well as their cell-specific profiles.

Our study highlights both the structural similarities and subtype-specific differences of NMDARs across various species and further validates the predicted structure of human NMDARs using biochemical methods. In addition, we predicted the binding modes of several small molecules and performed molecular dynamics simulations to investigate their interactions detail. Together, these findings offer new insights into the structural diversity of NMDAR subtypes and provide valuable clues regarding potential binding pockets, which may facilitate future small-molecule drug discovery and optimization.

## 2 Results

### 2.1 Spatial-temporal expression pattern of NMDARs in the brain

**Fig. 1:**
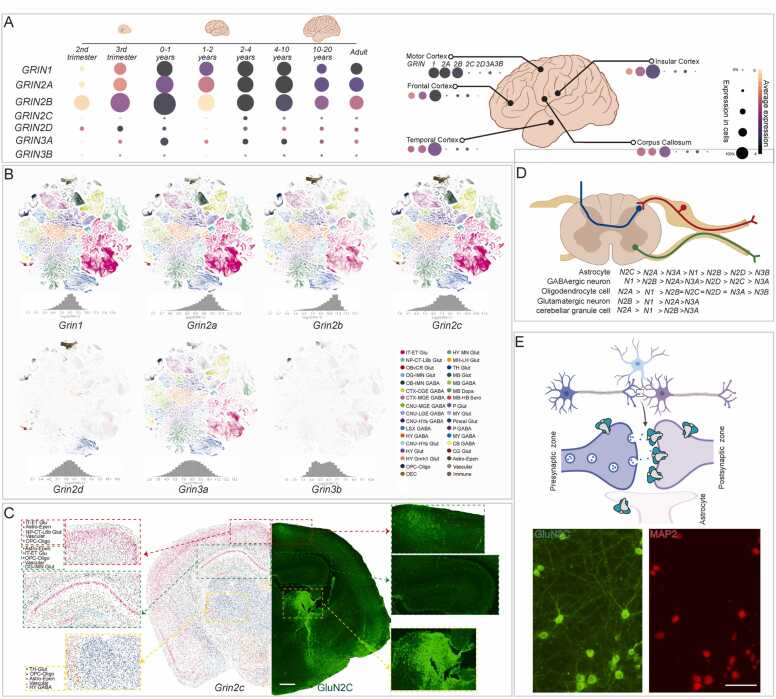
Spatial-temporal expression pattern of NMDAR subunits in the brain. (A) Spatial and temporal expression pattern of NMDAR subunits RNA during human brain development. (B) Neural subclasses containing clusters that express NMDAR subunits RNA. (C) Representative MERFISH (Multiplexed Error Robust Fluorescence in Situ Hybridization) section and immunostaining of GluN2C expression. (D) The expression pattern of NMDAR subunits RNA in the spinal cord. (E) Representative immunostaining of GluN2C with a neuronal somatodendritic marker MAP2 in cultured neurons.

Before structure prediction, we analyzed single-cell RNA sequencing data to explore the expression patterns of NMDAR subunits across human brain development using the UCSC Cell Browser [21]. GluN1, GluN2A, and GluN2B were consistently the most highly expressed subunits, with GluN2D expression also observed during early development. GluN3A showed high expression in infancy, whereas GluN2C and GluN3B expression remained low throughout all stages (Fig. 1A).

From the second trimester to infancy (0–1 year), the overall NMDAR subunit expression increased, peaking with GluN2B. This was followed by a sharp decline in GluN2B levels, which is consistent with the developmental switch to GluN2A-containing NMDARs [22]. This peak also corresponds to the maximal synaptic density, after which synaptic pruning occurs - a process potentially involving GluN3A and other subunits. During adolescence, expression levels stabilized, and after age 4-10 years, all subunits gradually declined except GluN2D (Fig. 1A).

We then explored the expression patterns in adult mouse brain regions. In higher-order brain regions such as the motor, frontal, temporal, and insular cortices, as well as in the corpus callosum [23] - a region known for high NMDAR expression - GluN1, GluN2A, and GluN2B were expressed at similar levels, with most cells showing high expression of these subunits. In the other four brain regions, GluN2B remained the most highly expressed subunit, followed by GluN2A and GluN1. The other four subunits were expressed at much lower levels, with GluN3A being more abundant than GluN2D, while GluN2C and GluN3B were barely detectable(Fig. 1A).

We further explored the expression of NMDAR subunits across different cell types in the brain based on the Allen Brain Cell Atlas. NMDAR expression was significantly higher in neurons compared to glial cells. Furthermore, excitatory neurons exhibited higher expression probabilities than inhibitory neurons (Fig. 1B). The expression levels of most subunits followed a near-normal distribution, except for the GluN3B subunit, which showed a bimodal distribution pattern (Fig. 1B).

Next, we mapped the expression of NMDAR subunits across different cell types in coronal adult mouse brain sections. We focused on three regions previously reported to show high GluN2C expression - the cortex [24], hippocampus [25], and striatum [26],and found that the IT-ET Glu neuron, Astro-Epen neuron, and Th-Glut neuron were the most GluN2C expressed cell type (Fig. 1C). To validate the single-cell data, we performed immunofluorescence staining. Consistent with the single-cell RNA data, GluN2C was expressed in the cortex, hippocampus, and striatum, with particularly strong expression in the striatum (Fig. 1C).

In the spinal cord, NMDAR subunits expression followed a pattern similar to that observed in the brain, with high expression of GluN2B, GluN2A, and GluN1 in astrocytes, GABAergic neurons, oligodendrocytes, glutamatergic neurons, and cerebellar granule cells (Fig. 1D).

To study the subcellular expression pattern of GluN2C in neurons, we used GluN2C antibodies to co-stain with a neuronal somatodendritic marker MAP2. Some GluN2C signals overlapped with MAP2, while others did not (Fig. 1E), suggesting that this subunit is expressed in both presynaptic and postsynaptic compartments. This is consistent with electrophysiological evidence that supports NMDARs play role in both pre- and post-synaptic activity [27].

These findings show that NMDAR subunits are widely and diversely distributed throughout the brain. The presentation of different cell types expressing different kinds of NMDAR subunits at different times in the same brain region also suggests the possibility of forming multiple subtypes of NMDARs.

### 2.2 NMDAR structural conservation in evolutionary history

To explore the evolutionary structural patterns of different NMDAR subtypes across species, we employed the AlphaFold multimer [17] and AlphaFold3 algorithms for structural predictions. We selected representative organisms for structural predictions, including fruit fly (*Drosophila melanogaster*), frog (*Xenopus laevis*), zebrafish (*Danio rerio*), mouse (*Mus musculus*), pig (*Sus scrofa domesticus*), chimpanzee (*Pan troglodytes*), and humans (*Homo sapiens*) (Supplementary Figure 3).

**Fig. 2:**
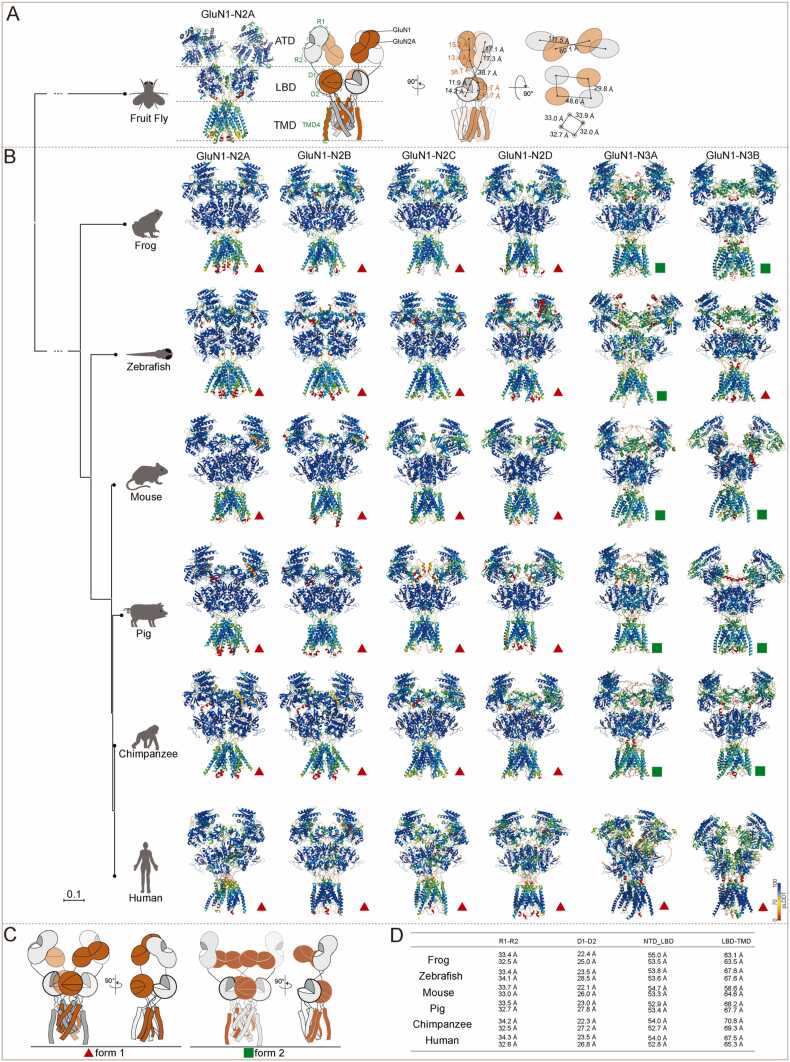
Prediction of NMDAR subtype structures across species using the AlphaFold2 or AlphaFold3. (A) Model of fruit fly NMDARs. (B) Model of NMDA subtypes across species. The receptors were associated in two forms that marked in red triangles (form 1) and green rectangles (form 2). (C) Cartoon models of form 1 and form 2 in (B). (D) COM distance of R1-R2, D1-D, ATD-LBD, and LBD-TMD of GluN1-N2A across species. All models are colored by confidence level (pLDDT) from very low confidence (red) to good confidence (yellow) to high confidence (blue).

For each prediction, we used the PLDDT score to assess the confidence of the predicted structures. A PLDDT score above 90 is considered excellent, 70–90 is acceptable, and below 70 requires careful evaluation before drawing conclusions. Similar to previously reported [28], all predictions maintained relatively high PLDDT scores, especially in the amino-terminal domains (ATD) and ligand-binding domain (LBD). Surprisingly, the transmembrane domain (TMD) also exhibited a relatively high PLDDT, prompting us to conduct a more detailed analysis. The domains with lower PLDDT scores appeared at the beginning of the ATD, the end of the TMD, and within the C-terminal domain (CTD). In particular, CTD had PLDDT scores consistently below 60 %, so we did not perform an analysis of this region ( Figs. 2A, 2B).

Overall, NMDARs across all species displayed a similar tetrameric bouquet-like structure, consisting of four layers of ATD, LBD, TMD, and CTD. For the fruit fly, the single NMDAR expressed forms a classic dimer-of-dimer configuration. During the transition from the ATD to the LBD, domain swapping occurs, although it took place at the dimer level rather than the tetramer level, as seen in higher organisms and our predictions of vertebrate NMDARs. Despite this difference, the internal arrangement of the ATD and LBD is similar to traditional NMDARs. Compared to GluN2, the GluN1 subunit's ATD exhibits a looser R1-R2 interface, with a 5.8 Å (34.4 Å vs. 28.6 Å) longer centers of mass (COM). However, the LBD of N1 and N2A remains comparable (26.1 Å vs. 27.4 Å). The transition from the ATD to the LBD in both subunits is consistent. In terms of cross-sectional structure, the ATD of the fruit fly also maintains GluN1 on the periphery and GluN2A in the interior, with the COM distance between the two GluN2A subunits being roughly half that between the two GluN1 subunits distance (Fig. 2A).

Starting with frog, seven subunits have been expressed and potentially form six di-heterotetrametric NMDARs: GluN1-N2A, GluN1-N2B, GluN1-N2C, GluN1-N2D, GluN1-N3A, and GluN1-N3B. Structurally, these subtypes can be divided into two major forms. All four GluN1-N2 subtypes in form 1 share a similar configuration, which is conserved across species from frogs to higher animals. Interestingly, this configuration shows remarkable structural conservation across species, as reflected by small root mean square deviation (RMSD) values. Moreover, the distance between the R1-R2 in the N1 subunits of GluN1-N2A across species ranges from 33.4 Å to 34.3 Å, while in the N2A subunits, the range is from 32.5 Å to 33.0 Å. Similar trends were observed in the COM distances between the D1-D2 regions of the LBD. Moreover, the distances between the ATD and LBD (52.9 Å to 55.0 Å for GluN1, 52.7 Å to 53,6 Å for GluN2A), as well as from the LBD to TMD4 (58.6 Å to 70.8 Å for GluN1, 63.5 Å to 69.3 Å for GluN2A), showed some variation (Figs. 2B, 2D).

All predicted GluN1-N2 receptors from both AlphaFold2 and AlphaFold3 predictions presented the conventional NMDA receptor conformation (Figs. 2B, 2C, form 1), consistent with previous cryo-EM and crystallography results. However, a new conformation was observed when the AlphaFold3 algorithm was used to predict GluN1-N3 type NMDARs (Figs. 2B, 2C, form 2). Specifically, under the AlphaFold3 algorithm, all GluN1-N3 NMDARs, except for the mouse GluN1-GluN3B, assembled in form 2. As noted in our previous studies [28], this assembly mode positions the ATD of GluN1 more vertically relative to the cell membrane, while the ATD of GluN3 lies more parallel to the membrane. This results in a nearly 90° angle between the R1-R2 axes of the ATD (Figs. 2B, 2C). Since we have previously assessed the validity of these two arrangements in humans [28], we will not elaborate further here. For non-human species, due to the lack of biochemical validation, we present the AlphaFold3 models without further experimental support.

The structural biology data and comparisons above indicate that the overall shape of the NMDAR is highly conserved across different species. In GluN3-related receptors, there appear to be two significantly distinct structural models. The human GluN1-N3A model [28], [29] and rat GluN1-N2A-N3A model (PDB: 8JF7), which align more closely with the traditional GluN1-N2 type, has been experimentally confirmed, while the other models remain less well-studied.

### 2.3 Structural homogeneity between different NMDAR subtypes

To assess the accuracy of the predicted NMDAR structures, we compared the human GluN1-N2 subtypes with the experimentally determined cryo-EM structures. The following cryo-EM models were used for comparison: GluN1-N2A (PDB: 7EOS), GluN1-N2B (PDB: 7EU8), GluN1-N2C (PDB: 8E92), and GluN1-N2D (PDB: 8E96).

**Fig. 3:**
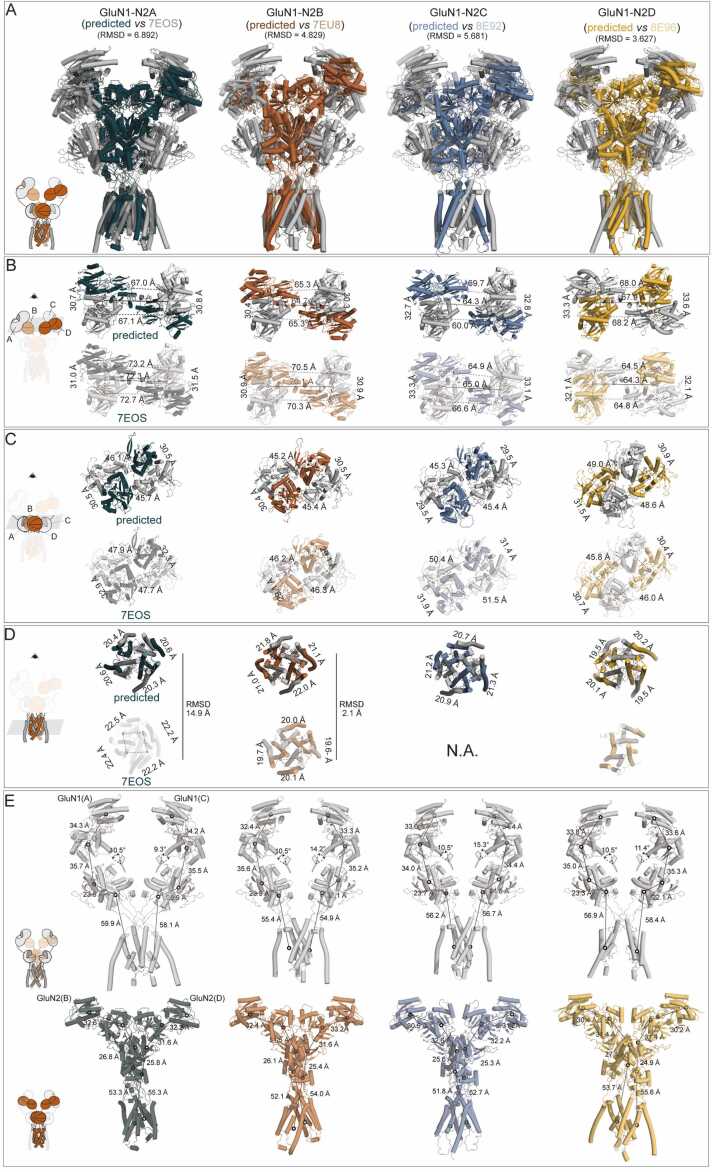
Comparison of predicted and cryo-EM revealed GluN1-N2 structures. (A) Superimposed predicted models and cryo-EM determined GluN1-N2A, GluN1-N2B, GluN1-N2C, and GluN1-N2D structures. (B-D) Cross comparation predicted models with cryo-EM determined GluN1-N2 structures at ATD (B), LBD (C), and TMD (D) layers. (E) Comparation of GluN1-N1 dimer and GluN2-N2 dimer between predicted models with cryo-EM determined GluN1-N2 structures.

The predicted models closely matched the cryo-EM data, with low overall RMSDs ranging from 3.63 Å to 6.90 Å (Fig. 3A). All subtypes exhibited a dimer-of-dimers arrangement in both the ATD and LBD regions. At the ATD level, the predicted GluN1-N2A and GluN1-N2B structures appeared more compact than the cryo-EM models. In contrast, GluN1-N2C and GluN1-N2D were predicted to be more flexible and less compact than their experimental counterparts (Fig. 3B).

The LBD, a stable region of NMDARs, was more accurately predicted. Except for GluN1-N2D, the predicted LBD structures were more compact than those from cryo-EM, both within the dimer and across dimers. For GluN1-N2D, however, the predicted model showed larger inter-dimer COM distances compared than the cryo-EM model (49.0 Å vs. 48.6 Å and 45.8 Å vs. 46.0 Å), while intra-dimer distances were consistent (30.9 Å vs. 31.5 Å and 30.4 Å vs. 30.7 Å) (Fig. 3C).

At the TMD level, the COM distances in GluN1-N2A were significantly larger in the experimental structure compared to the predicted one (89.3 Å vs. 81.9 Å), whereas the opposite was observed for GluN1-N2B (79.4 Å vs. 85.9 Å). Due to limited resolution of the TMD in the cryo-EM structures for GluN1-N2C and GluN1-N2D, a direct comparison for these subtypes was not possible. Nevertheless, across all four predicted subtypes, the TMD COM distances were nearly identical (Fig. 3D). To further assess structural differences, we evaluated the RMSD of the TMDs between the predicted and experimentally resolved models. The RMSD was 14.9 Å for GluN1-N2A, while for GluN1-N2B it was much lower at 2.1 Å. Considering the intrinsic flexibility of the TMD, its dependence on ligand binding, and the influence of cryo-EM map quality and model building strategies, we believe such variation in RMSD is justifiable and reflects biologically relevant conformational diversity.

Further analysis of the domain organization in the predicted models revealed nearly identical subunit arrangements, with slight differences in the orientation of domains. This resulted in a minor asymmetry across subtypes. For example, in GluN1-N2A, the R1-R1 COM distance between GluN1(A) subunits was 34.3 Å, while the LBD D1-D2 distance was 23.6 Å. Similar patterns were observed in GluN1-N2B, GluN1-N2C, and GluN1-N2D. The dihedral angle differences between the ATD and LBD were minimal for GluN1-N2A and GluN1-N2D (1.2° and 0.9°, respectively), while GluN1-N2B and GluN1-N2C showed greater asymmetry (3.8° and 4.8°), in line with the observed asymmetry in the cryo-EM structure of GluN1-N2C. Similar asymmetry was noted in the GluN2 subunits across all four NMDAR types (Fig. 3E).

In summary, these results demonstrate that the predicted and experimental structures maintain a considerable degree of consistency, suggesting the accuracy of the predictions. At the same time, the predicted structure provides information in the TMD region of GluN1-N2C and GluN1-N2D that the experimental structure does not fully provide.

### 2.4 Subunits arrangement and validation of GluN1-N2 NMDARs

**Fig. 4:**
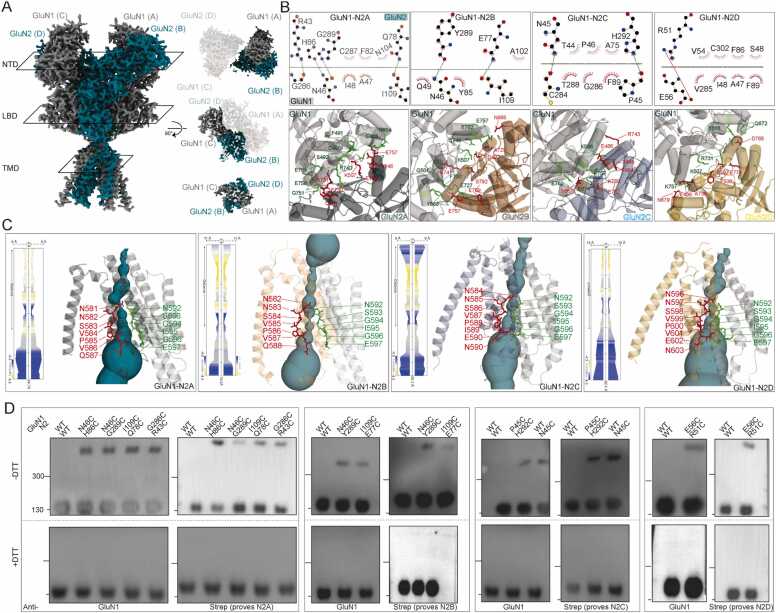
Subunit arrangements and interrelationships of the GluN1-N2 NMDARs. (A) Overall density model and subunit arrangement model at ATD, LBD, TMD layers of predicted GluN1-N2A. (B) Ligplot^+^ analysis of the R1-R1 interaction of the GluN1-N2 NMDARs. (C) Model of GluN1-N2 transmembrane domain. One of GluN1 and GluN2 subunits were transparent for clarification. View of a solvent-accessible surface carved along the pore axis using the MOLE. (D) Representative western blot results of detection of disulfide bonds by antibodies prove GluN1 and GluN2 in reducing (+DTT) and non-reducing conditions (-DTT), respectively.

To investigate the key amino acids involved in tetramer assembly, we conducted a more detailed analysis of the predicted models. Using LigPlot^+^ [30], we identified that the amino acids crucial for maintaining the ATD dimer assembly in GluN1 are primarily distributed between P45 and E56, with additional involvement from Y85, F89, I109, and C284-G286. In GluN1-N2A, important amino acid pairs such as N46 (GluN1) -H89 (GluN2A), N46-G286, I109-Q78, and G286-R43 facilitate ATD assembly by forming hydrogen bonds. Similarly, in GluN1-N2B, pairs like N46 (GluN1) -Y289 (GluN2B) and I109-E77 play comparable roles. For GluN1-N2C, the crucial residues are P45 (GluN1) -H292 (GluN2C) and C284-N45, while in GluN1-N2D, E56 (GluN1) -R51 (GluN2D) stabilize the ATD (Figs. 4A, 4B).

We also found that several amino acids in GluN1 are involved in forming the GluN1-N2 LBD dimer. In GluN2A, the residues contributing to LBD assembly include C501, K507, F730, R731, E762, Q746, and E757, while in GluN2B, the key residues are D492, N665, A725, E757, and E769. For GluN2C, the relevant residues are E486, F494, S495, N667, R743, K760, and E762, whereas in GluN2D, they are E498, F506, S507, N679, E774, and D768 (Figs. 4A, 4B).

We used MOLE [31] to analyze the GluN1-N2 channels. This region is challenging to study using traditional structural biology techniques because of the high degree of flexibility and embedded in the detergent. We observed strong conservation of the amino acids involved in the channel within GluN1, particularly amino acids from N592 to E597. In GluN2, the ‘NNSVP’ sequence is highly conserved and contributes to pore formation. Notably, the pore-forming sequences are identical between GluN2A and GluN2B, while GluN2C and GluN2D differ by only a single amino acid (‘IEN’ vs. ‘VEN’). The narrowest points of the GluN1-N2A pore are at N581 and N592, while for GluN1-N2B, they are at S584 and G594. In GluN1-N2C, the narrowest regions are N584-N592, and in GluN1-N2D, they are N596-N592 (Fig.4C).

We validated the predicted models through cysteine crosslinking experiments. Mutations were introduced at key amino acids involved in ATD assembly, given the ATD’s mobility and tolerance to crosslinking. For GluN1-N2A, we mutated four amino acid pairs, including N46C-H86C, N64C-G289C, I109C-Q78C, and G286C-R43C (Fig. 4B), all mediated crosslinking, as indicated by dimer bands in non-reducing western blots. Similarly, N46C-Y289 and I109C-E77C in GluN1-N2B, and R45C-H292C and WT-N45C in GluN1-N2C, successfully crosslinked. Additionally, E56C-R51C validated the GluN1-N2D model (Fig. 4D).

In summary, the above results identify the key amino acids that make up the tetramer at the ATD and LBD levels and further validate these amino acids by using traditional biochemical methods. Moreover, we also identify the pore-forming amino acids.

### 2.5 Binding niches of agonists and competitive antagonists

One major advantage of artificial intelligence algorithms is their ability to accurately predict protein-ligand binding interactions. In this study, we employed three of the latest algorithms, RoseTTAFold All-Atom, DiffDock-L, and DynamicBind for the prediction of the small molecular binding.

**Fig. 5:**
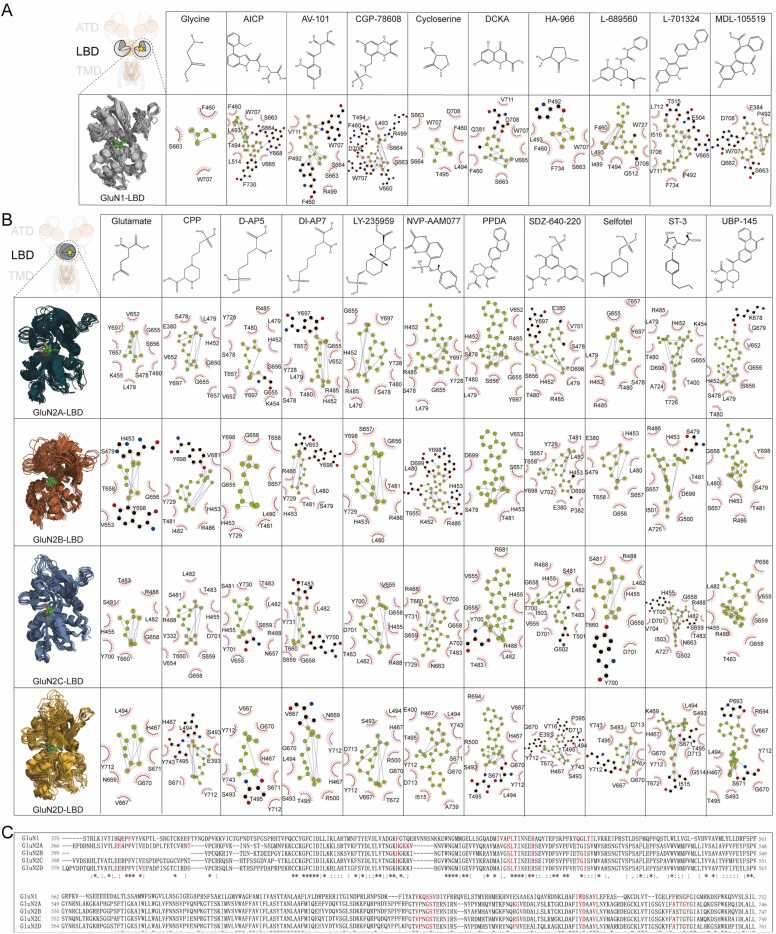
Prediction of the structure of small molecules bound LBD. (A) Chemical structure of small molecules, model illustration and Ligplot^+^ analysis of the binding interface of GluN1-LBD in complex of small molecules. (B) Chemical structure of small molecules, model illustration and Ligplot^+^ analysis of binding interface of GluN2-LBD complex of small molecules. (C) Sequence alignment of LBD domain of NMDAR subunits. The red marked amino acids are participating in the agonists and antagonists binding.

For the obligatory subunit GluN1, in addition to its endogenous agonist glycine, we also selected AICP [32], AV-101 [33], CGP-78608 [28], D-cycloserine [32], DCKA [34], HAP-966 [35], L689560 [36], L-701324 [37], and MDL-105519 [38] for prediction (Fig. 5A). These molecules serve various pharmacological roles. For instance, AICP is a GluN1-N2C–selective ligand and has been used as a tool to differentiate NMDAR subtypes. AV-101 has demonstrated therapeutic potential in animal models, such as alleviating dyskinesias in MPTP-treated monkeys. In contrast, HAP-966 has been shown to impair visual memory, illustrating the diverse functional consequences of GluN1-targeting ligands.

For the alternative subunit GluN2, besides the endogenous agonist glutamate, we included CPP [39], [40], D-AP5 [41], DL-AP7 [42], LY235959 [43], NVP-AAM077 [44], PPDA [45], SDZ-640–220 [46], [47], Selfotel [48], [49], [50], ST3 [51], and UBP-145 [52]. These compounds represent a wide range of functional activities. For example, several (e.g., CPP, D-AP5) exhibit antidepressant properties, while others, such as SDZ-640–220, have shown the ability to prevent synaptic toxicity. This selection of ligands provides a comprehensive set for evaluating subtype-specific binding and functional modulation of the NMDARs.(Fig. 5B).

Our analysis of the predicted protein-ligand complexes, carried out with LigPlot^+^, revealed that these small molecules primarily interacted through hydrophobic interactions and hydrogen bonds. For glycine binding, the key residues on GluN1 included F460, S663, and W707. Other small molecules bound to residues like Q381, F384, I488, P492, L493, T494, R499, Q512, T515, I516, as well as residues in the regions 660V-668Y, 706I-712L, F730, and F734. Notably, F460 and W707 in GluN1 were involved in almost all small molecule bindings (Fig. 5A).

In the case of GluN2, glutamate binding to GluN2A, GluN2C, and GluN2D was driven mainly by hydrophobic interactions, whereas in GluN2B, hydrogen bonds with H453 and Y698 were also observed (Fig. 5B). The specific binding niches for GluN2A involved residues H452, S478, L479, G650, G655, S656, T657, and Y697. In GluN2B, the key residues were H453, S479, G656, S657, T658, and Y698. GluN2C formed its binding niches through H455, S481, L482, T483, R488, G658, T660, and Y700, while in GluN2D, T465, H467, L494, T495, G670, and T672 were important.

Several regions of the small molecule binding pockets were conserved across all four GluN2 isoforms, including the sequences ‘SLTINEERSE’, TVPNGSTERNIR’, and ‘AFIYDAAVLN’. The shared conservation of LBD binding niches between the GluN1 and GluN2 subunits suggests a common evolutionary origin that may have been retained throughout development (Fig. 5C).

The results for small molecules binding to the obligatory subunit GluN1 and alternative subunit GluN2 showed highly consistent. Key binding residues for GluN1 ligands, including glycine and other small molecules, were primarily via hydrophobic and hydrogen bond interactions, with residues like F460 and W707 involved in nearly all ligand interactions. For GluN2 subunits, binding was also driven by hydrophobic interactions. Notably, binding regions of GluN2A, GluN2B, GluN2C, and GluN2D suggest evolutionary conservation of the binding sites.

### 2.6 Pore blocker binding cavities

The NMDA R's pore is essential to its conductivity and serves as a key target for pharmacological agents. In our study, we conducted docking experiments with 20 small molecules, utilizing RoseTTAFold All-Atom, DiffDock-L, and Dynamic bind to predict their binding interactions within the pore regions of GluN1-N2 NMDARs. This selection included clinically established drugs, such as memantine, ketamine, felbamate, and tramadol, as well as compounds that failed in trials like MK-801 and PCP. Some molecules, including CNS-1102, are still undergoing trials for their neuroprotective potential.

Our initial attempts using RoseTTAFold All-Atom to dock small molecules directly into the TMD were unsuccessful, likely due to limitations in generating high-quality TMD protein structures. The computational constraints and the complexity of predicting protein-small molecule interactions in this domain hindered further progress with this method. However, we achieved more accurate results with DiffDock-L and DynamicBind, which integrates AlphaFold prediction models to generate protein-small molecule complexes.

By focusing on the core helix of the TMD pore, we not only enhanced computational efficiency but also improved the accuracy of our predictions. In each DiffDock-L prediction, we generated 100–300 predictions, retaining only the highest-confidence structures for analysis. The confidence scores ranged from 0.11 to 4.11, with lower scores indicating higher prediction reliability. While the algorithm's precision is not absolute, and variability in predicted binding sites and dynamics exists, we prioritized the most confident predictions for further study. The small molecules were consistently predicted to bind within the vestibule region of the receptor pore, between the M3 helix bundle and the M2 pore loop (Supplementary Figure 1, ​Figure 2​), suggesting that they are in a pre-open or post-open metastable state.

Certain molecules, such as amantadine, kynurenic acid, 1-PCA, and Riluzole, demonstrated high confidence scores across different NMDAR subtypes. Others, like CNS-1102, showed lower confidence scores for all subtypes. These variations may be attributable to the inherent differences in the biophysical properties of the molecules and their interactions with the receptor, although our methods do not yet allow for precise predictions of binding affinities (Supplementary Figure 1).

**Fig. 6:**
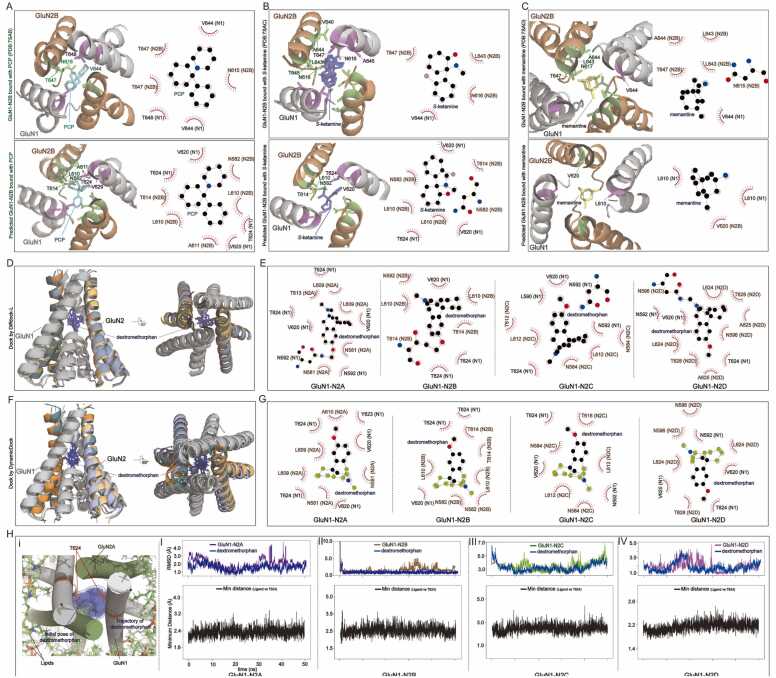
Comparison of predicted PCP, ketamine, and memantine bound GluN1-N2B models and their cryo-EM models. (A) Illustration and Ligplot^+^ analysis of PCP bound GluN1-N2B from both predicted and cryo-EM determined models. (B) Model illustration and Ligplot^+^ analysis of ketamine bound GluN1-N2B that from both predicted and cryo-EM determined models. (C) Model illustration and Ligplot^+^ analysis of memantine bound GluN1-N2B that from both predicted and cryo-EM determined model. (D, E) Model illustration and Ligplot^+^ analysis of dextromethorphan bound GluN1-N2 by DiffDock-L. (F, G) Model illustration and Ligplot^+^ analysis of dextromethorphan bound GluN1-N2 by DynamicBind. (H) MD simulation of dextromethorphan bound GluN1-N2. I-IV (up) RMSD trajectories for TMD of GluN1-N2 and dextromethorphan. (bottom) RMSD trajectories for the nearest distance of dextromethorphan to GluN1 T624.

We validated our docking predictions against cryo-EM-resolved structures of PCP, ketamine, and memantine, each of which binds within the pore region. For PCP, our predicted structure involved interactions with conserved residues GluN1 V620, T624, and GluN2B N582, L610, A611, and T614, with homology to residues identified in the cryo-EM structure (PDB 7SAB) (Fig. 6A). Similarly, ketamine was predicted to bind through interactions with GluN1 V620, T624, and GluN2B N582, L619, and T614, consistent with cryo-EM data (PDB 7SAC) (Fig. 6B, Supplementary Figure 2). Analyzing 7SAD we found that V644 of GluN1 and N615, L643, A644, and T647 of GluN2B are involved in memantine binding. In the predicted structure, L610 of GluN1 and V620 of GluN2B are involved in the binding (Fig. 6C). These results underscore the accuracy of our model in predicting small molecules binding to the NMDAR pore.

Due to limitations in computational resources, we were only able to perform detailed analysis on a subset of representative and functionally important compounds to validate our prediction results. Further analyses focused on dextromethorphan (DM), a commonly used cough suppressant [53] with neuroprotective effects but with risks of abuse [54], in different NMDAR subtypes. In GluN1-N2A, GluN1 N592, V620, and T624 were predicted to interact hydrophobically, along with GluN2A N581, L609, and T613. In GluN1-N2B, T614 of GluN2B formed a hydrogen bond, while in GluN1-N2C, interactions involved N592 of GluN1 and N584 and L612 of GluN2C. In GluN1-N2D, binding involved GluN1's N592 and T624 with GluN2D's N596, L624, A625, and T628 (Figs. 6D, 6E, Supplementary Figure 2).

To further validate the accuracy and reliability of these predictions, we employed DynamicBind, an AI-based tool with a protein-ligand interaction prediction performance comparable to that of DiffDock-L. As expected, both algorithms produced highly consistent binding pocket predictions for DM across the four NMDAR subtypes. Not only were the predicted binding sites located in similar regions of the transmembrane domain, but the involved amino acids also showed a high degree of overlap. For example, in the GluN1-N2A subtype, DiffDock-L predicted the DM binding pocket to include GluN1 N592, V620, and T624, and GluN2A N581, L609, and T613 (Figs. 6D, 6E). DynamicBind similarly predicted GluN1 V620, Y623, T624, and GluN2A N581, L609, and A620 (Figs. 6F, 6G). Considering the inherent flexibility of the transmembrane domain, the dynamic nature of small-molecule binding, and potential near-neighbor effects in AI predictions, the high degree of agreement between these algorithms provides strong support for the predicted DM binding sites. It is worth noting that in DiffDock-L prediction, in addition to generating some high-confidence positive predictions, it also generates many negative predictions (i.e., predicting that the binding site is not inside the channel, Supplementary Figure 2), whereas DynamicBind gets all the predictions positive.

To further assess the dynamic behavior of TMD and small molecular binding, we performed molecular dynamics (MD) simulations of the DM and GluN1-N2 complexes. Two simulations were conducted: a short MD simulation (50 ns, Fig. 6H) and a longer one (250 ns, Supplementary Figure 4). We first evaluated the stability of the system and the binding of DM to the receptor by analyzing both the protein RMSD and ligand RMSD. In both the short-term and long-term simulations, the RMSD values remained stable, indicating that the complex was well-equilibrated. Therefore, we proceeded with further analysis using the short simulation. As a key evaluation metric, we monitored the distance between DM and residue T624 of GluN1, as this residue was consistently predicted to interact with DM across all four NMDAR subtypes by both DiffDock-L and DynamicBind. As expected, T624 maintained a proximity to DM throughout the simulation, with the distance remaining mostly around 2.5 Å (Fig. 6H), further supporting the predicted binding interaction.

These results show that although the structures of the small molecules are very different, many of them share the same binding cavities and some even share similar amino acids. This not only reveals the binding mechanism of existing small molecules and summarizes the similarity pattern but also provides more amino acid resolution precise targets for subsequent drug development.

## 3 Discussion

Our study employed cutting-edge algorithms to predict the structures of NMDARs across different species, revealing significant evolutionary conservation in these receptors. By comparing these predicted structures with experimentally resolved human NMDAR structures, we demonstrated the high reliability of our predictions and filled in gaps in the existing structural data. Subsequently, we conducted a detailed analysis of the human GluN1-N2 receptor, identifying and mapping key amino acids involved in receptor assembly, and further validated through classical biochemical techniques. Additionally, using the RoseTTAFold All-Atom, we uncovered the highly conserved agonists and competitive antagonists binding pockets within different NMDAR subunits. Through DiffDock-L, DynamicBind, and MD simulation, we conducted molecular docking analysis on small molecules targeting TMD. Our findings offer new perspectives on the evolutionary conservation of NMDARs, the diversity of their distribution in the brain, structural specificity and conservation among subtypes, and the binding niches of small molecules.

NMDARs are fundamental to synaptic transmission, influencing critical functions such as learning, memory, and motor control [55]. They can be expressed at both pre- and post-synaptic sites, as well as in extra synaptic regions [56] and glial cells [4]. Aberrations in the function, expression, and distribution of NMDARs are implicated in various neurological and psychiatric diseases [57], [58]. Overactivation of these receptors can lead to excitotoxicity, contributing to abnormal neuronal death in several diseases [59]. Abnormally high expression or accumulation of NMDARs in specific types of post-synaptic neurons has been linked to mental health conditions such as depression [60]. Autoantibodies targeting NMDARs can trigger pathological receptor endocytosis, leading to conditions such as autoimmune encephalitis [7]. Therefore, understanding NMDARs is critical for both normal brain function and for addressing NMDAR-related diseases.

However, the NMDAR system is highly intricate and complex in nature. During early development, NMDARs and non-NMDARs diverge to achieve more refined neural regulation [9]. NMDARs are already expressed in organisms as early as Drosophila, and the differentiation of NMDAR subunits is observed in more evolved animals. Although there are significant differences in the protein sequences of NMDARs between lower and higher organisms, our structural analysis reveals a considerable degree of similarity in their 3D structures. These studies indicate that NMDARs share common structural features, such as a pseudo-fourfold symmetric "bouquet-like" architecture. Structurally, they are organized into ATD, LBD, TMD, and CTD. While we observed two distinct conformations in the analysis of the GluN1-N3 receptors, the underlying reasons remain unclear. Studies, others [29] and our cryo-EM data (PDB: 8JF7) and structural predictions [28], suggest that the human GluN1-N3 receptors tend to adopt the traditional NMDAR conformation, though this has not yet been reported in other species. In this study, by combining AI-based structural prediction with traditional biochemical cross-linking techniques, we obtained and validated the apo-state structure of the diheteromeric GluN1-N2 receptor. We further investigated the brain-region-specific expression patterns of NMDAR subunits using single-cell RNA sequencing (scRNA-seq) data. However, a significant gap remains between transcriptomic expression and the actual formation of functional NMDARs. For example, in certain cell types, the expression levels of GluN2A or GluN2B subunits were found to exceed that of GluN1, raising doubts about the assembly of functional diheteromeric receptors. Moreover, multiple layers of post-transcriptional regulation can influence receptor composition and function. These include the subunit combination strategy adopted by the cell - assembling diheteromeric or triheteromeric receptors - as well as the dynamic trafficking of NMDARs through tightly regulated endocytosis and exocytosis mechanisms.

Given the physiological significance of NMDARs, their subtype diversity, and the role of small molecules targeting these receptors, a variety of small molecules have been developed [55], [61]. These include competitive or non-competitive inhibitors targeting the agonist binding pockets within the LBD, pore-blockers targeting the TMD, and allosteric modulators without layer specificity. Despite these advancements, traditional structural biology has primarily focused on NMDARs from humans, rats, and a few chimeric models. Through these efforts, we have gained some understanding of the structure of several NMDAR subtypes in humans and identified small-molecule binding sites and key amino acid residues. However, traditional approaches have certain limitations. They often require large quantities of protein, even though cryo-EM reduces the demand compared to X-ray crystallography. Furthermore, some regions, particularly the TMD, remain difficult to resolve using conventional structural methods, making the structural characteristics of small molecules binding largely unknown.

The rise of artificial intelligence (AI) has provided new tools for studying the structural features of NMDARs. Notably, the Nobel Prize-winning AlphaFold [62] and RoseTTAFold [63] algorithms have enabled researchers to predict high-accuracy protein structures from sequences. RoseTTAFold All-Atom [18] and new-generation docking algorithms, such as DiffDock-L [19] and DynamicBind [20], allow researchers to provide personalized small molecules for *de novo* complex structure generation or dock them into structures calculated by other algorithms. These AI-driven methods empowered our research, enabling us to quickly and accurately compare NMDAR structures across different species and, more importantly, analyze multiple receptor-small molecule complexes. However, protein structure prediction algorithms still have limitations. For example, AI-based structure prediction methods cannot yet reliably distinguish between apo and holo states, or between active and various inactive conformations of proteins, thereby limiting their ability to capture dynamic structural transitions. Furthermore, while predictions based on homologous or known structures can achieve high accuracy, this level of precision is not guaranteed for previously uncharacterized proteins, even though such predictions are made in a model-free manner. Additionally, proteins are inherently dynamic and flexible, particularly in functionally critical regions such as the TMD of NMDARs. At present, AI algorithms can only provide structural snapshots rather than capturing the full spectrum of conformational dynamics.

To address these limitations, we not only compared and analyzed the predicted models against available experimental structures but also employed traditional biochemical approaches to further validate our findings. For predictions involving small-molecule binding sites - especially for TMD pore blockers - we performed molecular dynamic (MD) simulations to capture the dynamic nature of ligand binding and pocket flexibility. Ideally, electrophysiological validation or calcium imaging would be the gold standard to confirm functional relevance; however, due to current laboratory constraints, such experiments could not be conducted at this stage.

In summary, this study used state-of-the-art algorithms to predict the structures of NMDARs across different species, revealing both the conservation and diversity of these receptors across and within species. We identified and mapped the binding pockets and key amino acid residues for small molecules targeting the LBD and TMD of NMDARs. Our findings offer valuable insights into the widespread distribution and structural diversity of NMDARs, providing new perspectives for the development of small-molecule drugs targeting these receptors. These insights pave the way for the development of novel drugs and potential lead compounds.

## 4 Materials and methods

The management and utilization of animals were strictly in accordance with the requirements of the Experimental Animal Ethics Committee of the Second People's Hospital of Shenzhen (Approval number: 20240076).

### 4.1 Cell transfection and Western blotting

The human GluN1 (residue 1–847, NP_015566), GluN2A (residues 1–841, NP_000824), GluN2B (residues 1–842, NP_000825), GluN2C (residues 1–847, Q14957), GluN2D (residues 1–847, NM_000836) were used for the expression plasmids construction as previously described [28] with a small modification. The Strep tag was fused with CTD of GluN2. Targeted mutagenesis procedures were polymerase reactions using KOD Fx (Takara) on wild-type plasmids. The HEK293S cell was used for the PEI transfection. Cells were harvested 24–48 h post-transfection and resuspended in buffer containing 20 mM Tris-HCl (pH 7.4), 150 mM NaCl, 1 % lauryl maltose neopentyl glycol (LMNG), and protease inhibitor cocktail (Roche). Cells were lysed on ice for 30 min, during which time they were turned up and down 2–3 times to allow full lysis. After centrifugation at 15,000 g, the supernatant was subjected to SDS-polyacrylamide gel electrophoresis (10 % or 16 %) in the presence or absence of 100 mM DTT. The proteins were transferred to PVDF membranes. The membranes were blocked with TBST (20 mM Tris-HCl (pH 7.4), 150 mM NaCl, and 0.1 % Tween-20) containing 10 % milk and then incubated with mouse monoclonal antibodies against GluN1 (Abcam), Strep (Abcam), followed by HRP-conjugated anti-mouse or anti-rabbit antibodies (Proteintech). Protein bands on the membranes were visualized using a Western Bright™ reagent kit (Advansta).

### 4.2 Brain acquisition and immunofluorescence staining of brain sections

Brain section preparation and immunofluorescence staining were performed as previously described [64]. After anesthesia, the mice were perfused with 30–40 mL of saline through the left ventricle, followed by 30–40 mL of 4 % paraformaldehyde, and then fixed for 1 h before removing the brain. The brain was placed on ice and immersed in 4 % paraformaldehyde for about 4 ∼ 6 h. The brain was then transferred and submerged in 20 % sucrose solution, sunk and transferred to 30 % sucrose solution, and after the brain was sunk again, coronal frozen sections were made, and the brain slices were 30 μm thick. After that, immunofluorescence staining of brain sections was conducted. Briefly, the brain sections were washed with PBS and blocked with 10 % calf serum for 1 h, then sections were stained with GluN2C (Abcam) overnight at 4°C; After washed with PBS the sections were stained with anti-rabbit IgG-Alexa Fluor 488 (Abcam) at 37°C for 1 h. Brain slices were then washed with PBS and mounted using ProLong™ Gold anti-fade reagent (Thermo Fisher Scientific). After that brain slices were imaged by the VS120 microscope with excitation and emission wavelengths of 488 and 519 nm, respectively.

### 4.3 Hippocampal neuron culture and immunofluorescence staining

Cultured rat hippocampal neurons are detailed in our previous descriptions [61]. In brief, hippocampal tissue was obtained from Sprague Dawley rat embryos at approximately 17 days of pregnancy. After removal of the meninges, blood vessels, and cortex, freshly dissected hippocampi were cut into small pieces and adequately minced with surgical scissors, digested with 0.25 % trypsin at 37°C for 15–30 min, real-time observation of hippocampal tissue under the microscope every 5 min during digestion, and then the digestion was terminated by the addition of DMEM containing 10 % fetal bovine serum. The cells were filtered through a 200-mesh cell sieve and centrifuged at low speed (800 rpm/min) for 5 min to remove the supernatant. The precipitation is hippocampal neurons and cell resuspension was performed. Cells were plated at a density of 4 × 10^5^ cells/mL in a culture dish coated with poly (L-lysine) using DMEM containing 20 % fetal bovine serum. After the hippocampal neurons had almost finished attached (about 6 h after inoculation), the medium was replaced with neuron-specific neurobasal medium (Gibco) containing 2 % B27 supplement and changed every 72 h. The neurons used in the experiment were 14–21 days old. The neuron staining with GluN2C and MAP2 was performed as previously reported [7]. Briefly, cells cultured on slides were washed twice with PBS and fixed with 4 % paraformaldehyde for 5–10 min, after washed with PBS for 3 times, the cells were first incubated and blocked at 37 °C in PBS solution containing 10 % calf serum and 0.2 % Triton for 1 h; Then, cells were incubated with mouse-anti-GluN2C (Abcam) and rabbit anti-MAP2 (Abcam) overnight at 4°C, washed with PBS and then stained with anti-rabbit IgG-Alexa Fluor 488 and anti-mouse IgG-Alexa Fluor 594. Cells were washed with PBS and mounted using ProLong™ Gold antifade reagent. Images were imaged by the VS120 microscope at excitation and emission wavelengths of 488 and 519 nm and 591 and 618 nm, respectively.

### 4.4 Sc-RNA sequencing analysis and phylogenetic analysis

Publicly available online datasets (Allen brain map; https://singlecell.broadinstitute.org/single_cell) and the UCSC Cell Browser (UCSC Cell Browser) were conducted for single-cell RNA sequencing (sc-RNA seq) analysis. The GluN1 sequence of *Drosophila melanogaster* (NP_730940.1), *Xenopus laevis* (A0A1L8F5J9), *Danio rerio* (ENSDARP00000107703), *Mus musculus* (P35438.1), *Sus scrofa domesticus* (A0A287AWP0), *Pan troglodytes* (XP_024201759.1), *Homo sapiens* (A0A1L8F5J9.1) were used for the phylogenetic analysis. MEGA7 [65] was used for protein sequence alignment with default parameters, and the maximum likelihood (ML) method was used.

### 4.5 NMDARs structure prediction

The following full amino acid sequence of NMDAR subunits from difference spices were used: *Drosophila melanogaster* GluN1 (NP_730940.1) and GluN2 (NP_001284788.1); *Xenopus laevis* GluN1 (A0A1L8F5J9.1), GluN2A (B7ZSK1.1), GluN2B (A7XY94.1), GluN2C (ENSXETP00000014979), GluN2D (ENSXETP00000101091), GluN3A (ENSXETP00000020215), and GluN3B (ENSXETP00000056951); *Danio rerio* GluN1 (ENSDARP00000107703), GluN2A (ENSDARP00000116766), GluN2B (ENSDARP00000047093), GluN2C (ENSDARP00000127286), GluN2D (I3NI77), GluN3A (ENSDART00000109916.3), GluN3B (ENSDARP00000140721); *Mus musculus* GluN1 (P35438.1), GluN2A (P35436.2), GluN2B (Q01097.3), GluN2C (Q01098.2), GluN2D (Q03391.3), GluN3A (A2AIR5.1), GluN3B (Q91ZU9.1); *Sus scrofa domesticus* GluN1 (A0A287AWP0), GluN2A (A0A287BN68), GluN2B (A0A4X1V2X7), GluN2C (A0A4X1SIG1), GluN2D (F1RL86), GluN3A (A0A4X1VH63), GluN3B (3LMD3). *Pan troglodytes* GluN1 (XP_024201759.1), GluN2A (XP_024201759.1), GluN2B (XP_016778489.1), GluN2C (XP_016788369.2), GluN2D (XP_054529662.1), GluN3A (PNI49247.1), GluN3B (XP_024207521.2). *Homo sapiens* GluN1 (Q05586.1), GluN2A (Q12879.1), GluN2B (Q13224.3), GluN2C (Q14957.3), GluN2D (Q15399.2), GluN3A (Q9R1M7.1), GluN3A (O60391.2). The NMDARs of Drosophila melanogaster, Xenopus, Zebrafish, Mouse, Pig, and Chimpanzee were predicted by AlphaFold3. The Human NMDARs were predicted by Colab version of AlphaFold2 [66]. Structures of subunits were found in AlphaFold Protein Structure Database (https://alphafold.ebi.ac.uk/). No templates were used for any of the predictions. For Alphafold3, all parameters were set as default. For Alphafold2, setting was set as follows: max_seq = 508, max_extra_seq = 2048, number of recycle = 2, number of models = 2, use amber to relax. Four 4090 GPUs from Matpool (https://matpool.com/) were used to predict the structure of these structures. It took about 5 days to predict the first NMDA structure, and then about 20 h to predict the other structures.

### 4.6 Prediction of protein-ligand complex structure

For small molecules binding, we used both locally installed RoseTTAFold All-Atom [18] and online services. To reduce the computer burden, for the prediction of LBD in complex of small molecules, we used the ATD truncated GluN1 (371−821), GluN2A (367−809), GluN2B (366−820), GluN2C (369−822), and GluN2D (376−831). All parameters were set as default and above PDB files and small molecular sdf files were as inputs. Only one model with the highest confidence was produced. It took around one hour for a typical prediction with one 4090 TI GPU workstation.

For the prediction of TMD in complex small molecules, we used location installed or online services of DiffDock-L and DynamicBind. To reduce the computational burden, we used the core domain of TMD of GluN1-N2A, GluN1-N2B, GluN1-N2C, GluN1-N2D that basically contained the transmembrane domain 2 and transmembrane domain 3 and the linker between them. All parameters were set as default and above PDB files and small molecular sdf files were as inputs. One hundred to three hundred (DiffDock L) or ten (DynamicBind) different predictions will be produced and list by confidence score. The one binding to channel pore with highest score was used for analysis. It took around two hours for a typical prediction with one 4090 TI GPU workstation. All the SDF files were either downloaded from PubChem or produced by ChemDraw (V22).

### 4.7 MD simulation

The predicted GluN1-N2 and dextromethorphan (DM) models were used as the initial models for molecular dynamics. The initial model was embedded in a pre-equilibrated palmitoyl oleoyl phosphatidylcholine (POPC) bilayer containing ∼70 lipid molecules using CHARMM-GUI [67]. The generated system was solved by TIP3P waters and neutralized with 0.15 M NaCl, leading to ∼22,000 atoms.

The parameter files for MD simulations were prepared and downloaded from the CHARMM-GUI website. With a series of restraints for the proteins, ligands, and lipid atoms, the solvated system was subjected to energy minimization. Then the minimized systems were heated from 0 to 310 K and equilibrated at constant pressure and temperature (NPT ensemble; 310 K, 1 bar) by gradually decreasing the positional restraints on the protein, ligand, and lipid atoms. The MD simulations were performed with the Gromacs 2024 program package [68] using CHARMM36 force field. The results of the MD simulations were analyzed by Gromacs tools and VMD.

## CRediT authorship contribution statement

**Zengwei Kou:** Writing – original draft, Visualization, Investigation, Formal analysis, Conceptualization. **Dan Li:** Investigation, Conceptualization. **Han Tang:** Investigation. **Yunsheng Liu:** Writing – original draft, Investigation. **Jinfang Zhang:** Investigation.

## Declaration of Competing Interest

The authors declare that there is no conflict of interest.

## Footnotes

Appendix A Supplementary data associated with this article can be found in the online version at doi:10.1016/j.csbj.2025.07.027.
