# Supplementary material for: Artificial intelligence insight on structural basis and small molecule binding niches of NMDA receptor
Source: Comput Struct Biotechnol J. 2025 Jul 14;27:3167–80. doi: 10.1016/j.csbj.2025.07.027 (PMC12302824; doi:10.1016/j.csbj.2025.07.027)
Supplement: Supplementary file 1 — Supplementary material [file mmc1.docx]

**Supplementary Figure**

**Supplementary Figure 1. Prediction of the structures of pore blocker bound TMD**

Chemical structure of small molecules and their models that bind to TMD.

**~~
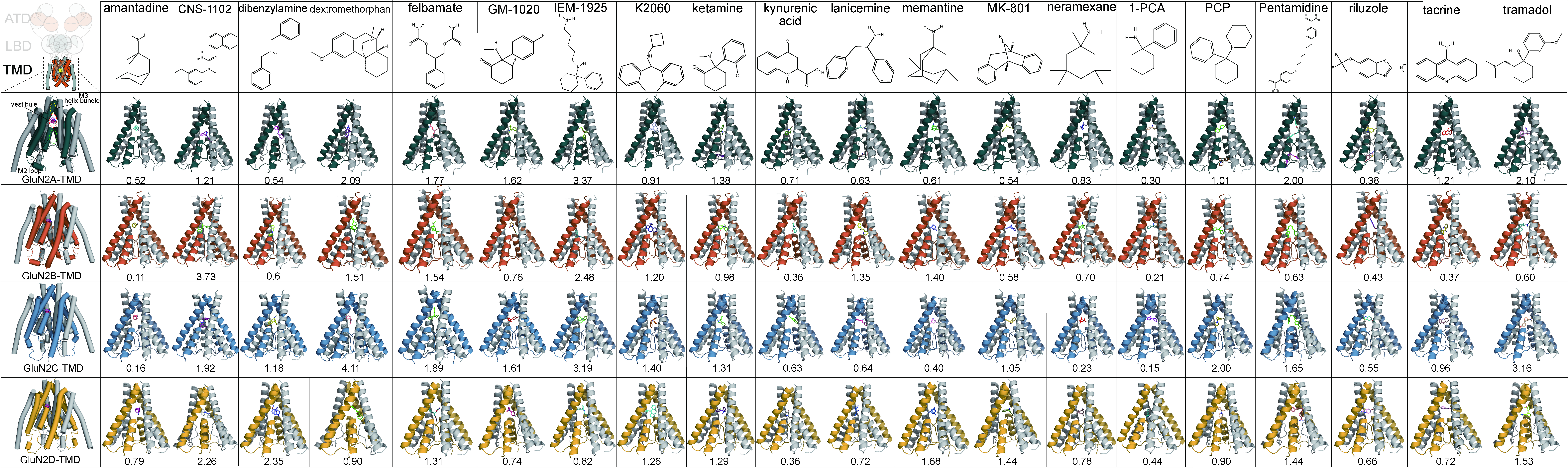
~~**


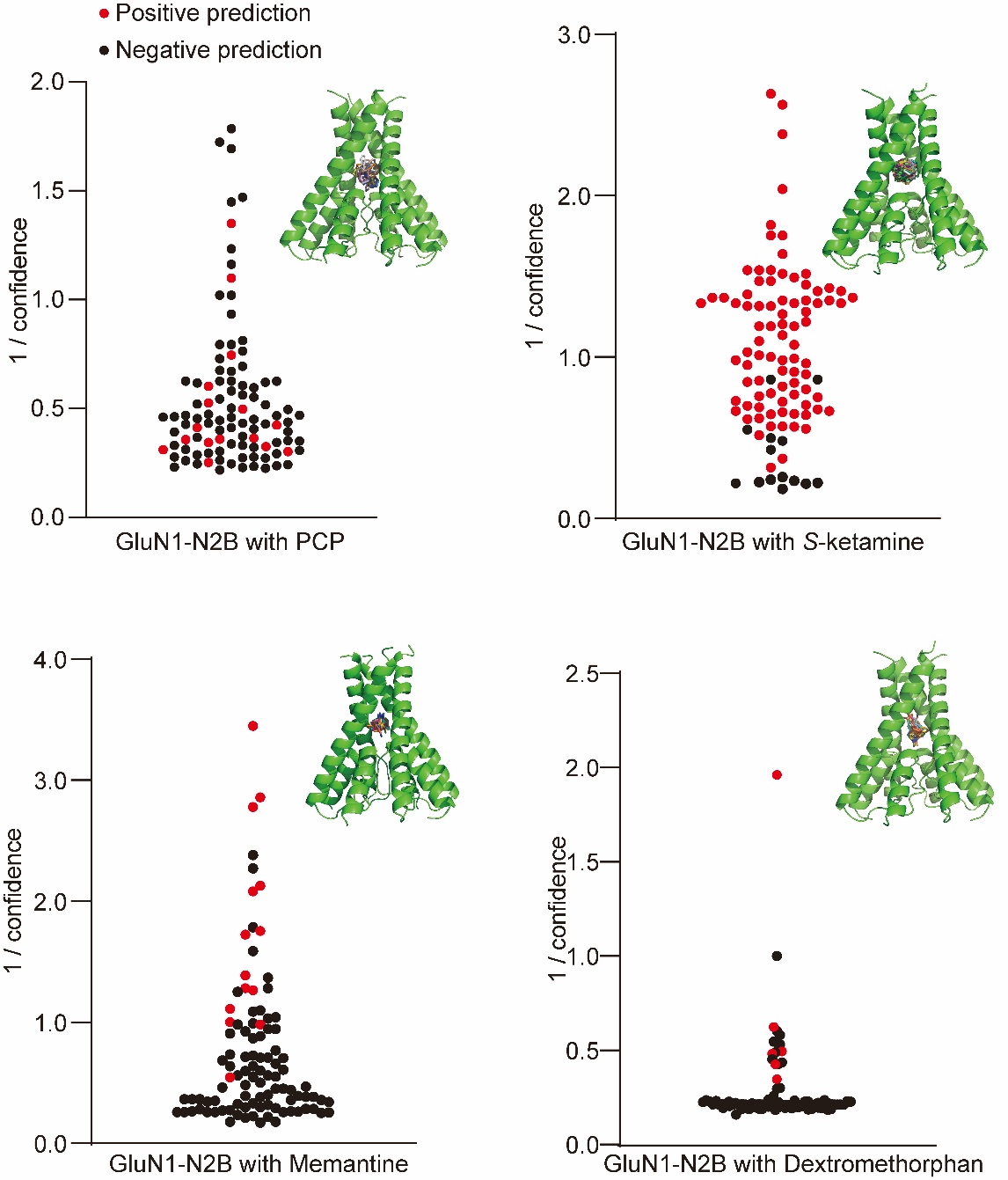


**Supplementary Figure 2. Plot of confidence from DiffDock-L prediction of GluN1-N2B in complex of PCP, *S*-ketamine, Memantine, and Dextromethorphan. The inserts show merged positive predictions.**

**
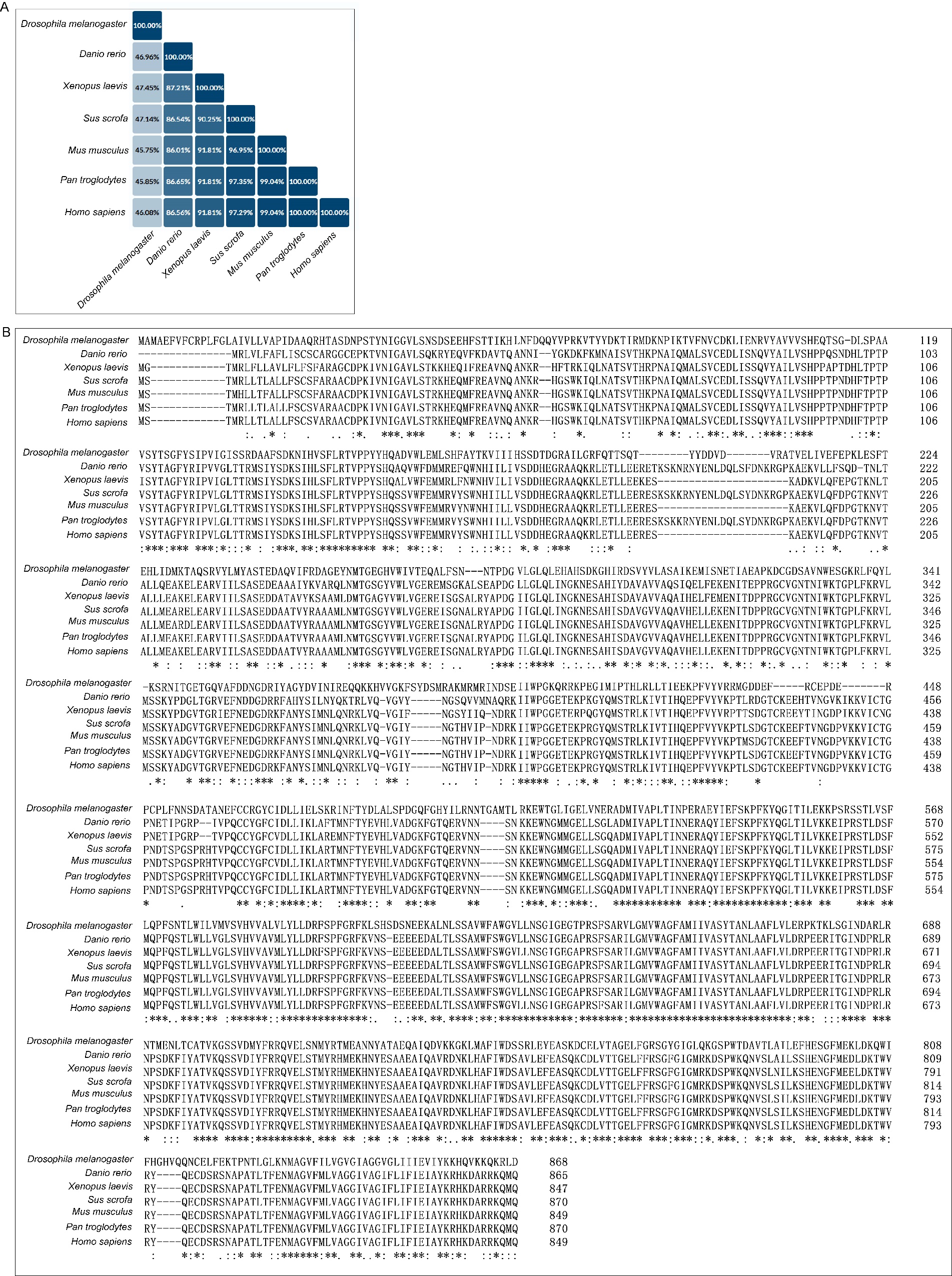
**

**Supplementary Figure 3. Percent identity matrix(A) and sequence alignment of GluN1 in different species(B).**

**
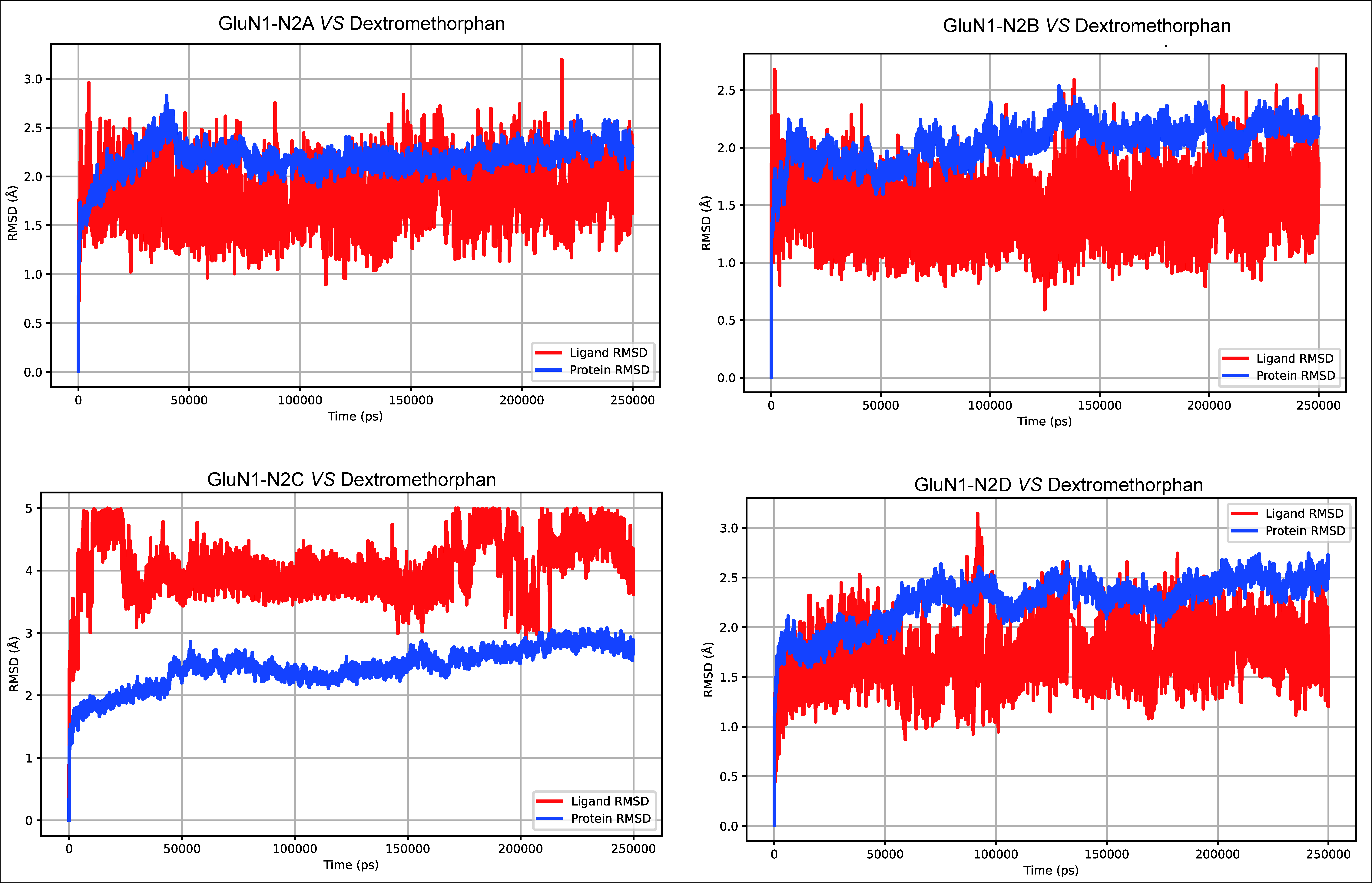
**

**Supplementary Figure 4. The 250 ns MD simulation of dextromethorphan-bound GluN1-N2. RMSD trajectories for TMD of GluN1-N2 (Protein) and dextromethorphan (Ligand).**
